# Machine learning-based risk stratification of early graft failure in simultaneous pancreas–kidney transplantation

**DOI:** 10.1038/s41598-026-47730-0

**Published:** 2026-04-04

**Authors:** Omar Altamimi, Hamza Nabulsi, Majdi Al-Shehab, Yousef Abbasi

**Affiliations:** The University of Jordan School of Medicine, Amman, Jordan

**Keywords:** Diseases, Endocrinology, Health care, Medical research, Nephrology, Risk factors

## Abstract

Simultaneous pancreas–kidney transplantation (SPK) restores insulin independence for patients with diabetes and end-stage renal disease, yet early graft failure remains a major obstacle. We analyzed 6,725 adult SPK procedures recorded by UNOS between 2014 and 2024 to build a peri-operative risk model using Random Survival Forests (RSF). After preprocessing, MissForest imputation and LASSO screening, 21 predictors were retained. The RSF, trained on 80% of the data and tested on the remainder, achieved a Harrell C-index of 0.73 and a Uno C-index of 0.58; time-dependent AUCs were 0.75 and 0.74 at one and two years, with Brier scores of 0.075 and 0.090. Maximizing Youden’s J produced a risk-score threshold of 105.24 that separated recipients into high- and low-risk strata with strongly divergent Kaplan–Meier curves (log-rank *p* = 2.4e−16). Decision-curve analysis demonstrated net clinical benefit across threshold probabilities 0–0.25 and, at a 10% threshold, predicted 41 unnecessary interventions avoided per 100 patients relative to treating all. Pre-transplant insulin status, cold-ischemia time and recipient age dominated variable importance. A compact RSF based on routinely collected peri-operative data provided internally validated graft-failure risk stratification that may support peri-operative risk assessment and targeted surveillance. Prospective multi-center validation is required before clinical implementation.

## Introduction

Simultaneous Pancreas–Kidney (SPK) transplantation is generally regarded as the gold standard therapeutic option for patients suffering from type 1 diabetes coupled with ESRD (End-stage renal disease), this is due to its ability to restore complete function and remove insulin reliance, which provides a large quality of life improvement for patients.^1,2^.

In 2023 out of 915 pancreas transplants, over 800 were SPK which reflects its current dominance as the preferred method. Graft failure in SPK transplants is different for the pancreas and the kidney. For the pancreas, the 1-year graft failure rate was 9.2%, while 90-day graft failure was 6.4%, indicating that a substantial proportion of failures occur early after transplantation. Kidneys, on the other hand, were faced with a lower graft failure rate at 1-year of 3.8%^3^. These rates present a challenge that must be solved, ideally by tailoring therapies to those at most risk.

Personalized medicine is a broad term that covers the intersection of multi-omic data, medical history, and social history to precisely characterize or provide therapy for an affected individual^4^. Artificial intelligence has become a major player in personalized medicine in recent years, namely in solid organ transplantation^5^. Factors such as immune profiling and genetic predictors have been evaluated as potential measures for the prediction of graft survival^6,7^.

In this context, AI can serve as a personalized medicine tool by combining multiple donor, recipient, and procedural variables to generate individualized estimates of graft-failure risk. Unlike traditional regression models, machine-learning methods such as random survival forests can flexibly capture non-linear relationships and interactions between variables without requiring proportional-hazards assumptions, thereby potentially uncovering clinically relevant patterns that are not easily modeled using conventional approaches.

Several studies have evaluated survival in SPK transplantation. Owen et al. evaluated survival across multiple centers in the UK using Cox-regression, Xuan Ho et al. conducted a similar analysis on data from the UK Transplant Registry focusing on donor blood tests.^8,9^ Vigia et al. employed machine learning (Naïve Bayes and Support Vector Machines) to find features that could be used to identify at-risk patients.^10^.

This study applied a Random Survival Forest (RSF) methodology, which is a machine learning technique built on Random Trees but tuned to work with right-censored data.^11^ An important distinction between RSF and Cox-regression is that RSFs can account for the non-linearity in the interactions between features and the fact that it does not assume proportional hazards which is necessary for Cox-regression yet unrealistic in most applications.^12^ While Naïve Bayes and Support Vector Machines are machine learning algorithms, they are not capable of providing risk scores and survival functions which are the main output used in this analysis.

This study aimed to develop a peri-operative model for risk stratification of pancreas graft failure after SPK transplantation using routinely collected clinical data, with the goal of supporting follow-up planning and peri-transplant decision-making.

## Methods

### Data source and cohort selection

Data were obtained from the United Network for Organ Sharing (UNOS) registry, comprising transplant procedures performed in the United States. Adult recipients (aged ≥ 18 years) of simultaneous kidney-pancreas (SPK) transplants conducted between 2014 and 2024 were included. This period was selected to account for the Kidney Allocation System (KAS) implementation in late 2014, which specifically impacted kidney and SPK transplant allocation practices^13^. Exclusion criteria were: (1) recipients of living donor grafts, due to different associated variables; (2) pediatric recipients, representing < 2% of the sample; and (3) patients with missing survival outcome data.

Failure was defined as Pancreatic graft failure, which, according to OPTN guidelines, includes, (1) Removal of the transplanted organ, (2) Recipient death, (3) Re-registration for pancreas, (4) registration for an islet transplant after a pancreas transplant, (5) A recipient’s total insulin use is greater than or equal to 0.5 units/kg/day for a consecutive 90 days^14^.

### Data preprocessing and feature engineering

Data processing was performed using Python (version 3.11.4) and the pandas library (version 2.2.3). Features with > 40% missing values (*n* = 212) and near-zero variance (defined as > 95% identical values; *n* = 67) were removed.^15–17^ Additional feature reduction, informed by clinical relevance and the UNOS data dictionary, involved removing redundant (*n* = 60), clinically irrelevant (*n* = 49), and post-transplant variables (*n* = 18).

Two engineered features were created: years living with diabetes (calculated as the interval between the recorded age of onset of diabetes and the age at transplantation) and years under dialysis prior to transplant (calculated as the interval between dialysis initiation date and the transplant date). Source variables used for engineering were subsequently removed. After preprocessing, 66 features remained.

Prior to further feature selection and imputation, the dataset was randomly split into an 80% training set (*n* = 5,380) and a 20% testing set (*n* = 1,345), ensuring independence throughout model development.

All data-driven model-development steps, including missingness-indicator selection, imputation fitting, feature selection, hyperparameter optimization, and risk-threshold derivation, were performed using the training set only, while the test set was reserved exclusively for final evaluation.

### Handling of missing data

Selective Missing Indicator Method (SMIM), in which a flag is added into a separate column for each missing value then a log-rank test is applied for each column to determine the variables whose missingness is significantly correlated with the outcome i.e. relevant, was applied. Variables demonstrating significant differences (*p* < 0.05) between missing and non-missing groups had missingness indicators appended (*n* = 4: Venous Extension Graft, Insulin usage, Dialysis, and Peripheral vascular disease).

For variables whose missingness was significantly associated with outcome, binary missingness indicators were created, where ‘Missing’ denotes that the original variable was unavailable/uncaptured in the registry for that record.

Missing values were imputed using the MissForest algorithm (Python MissForest library, max_iter = 10), a non-parametric, random forest-based imputation method appropriate for mixed-type data under missing at random (MAR) assumptions. As demonstrated by Van Ness et al., this approach also partially addresses missing not at random (MNAR) scenarios^18,19^.

### Feature selection

Final feature selection was conducted on the training set only using a LASSO-penalized Cox proportional hazards model (CoxnetSurvivalAnalysis, scikit-survival library). Five-fold cross-validation was performed to select the optimal regularization parameter. Features with nonzero mean coefficients across folds were retained, resulting in 21 predictors.

No univariate pre-screening was applied prior to the LASSO-penalized Cox model.

### Model development

RSF hyperparameters were optimized via Bayesian optimization using the Optuna library, targeting maximum Uno’s C-index on the cross-validated training set.^11,20^ The final RSF model was trained on the full training set and evaluated on the blinded test set.

### Model evaluation

The discriminative ability of the model was assessed using both Harrell’s and Uno’s C-indices as the data had a high censoring percentage 81.7% on the training set and 82.6% on the test set.^21^ Time dependent AUCs were calculated at 1 and 2 years to assess the model’s predictive abilities at those time points, while Brier scores were calculated at those same time points to assess calibration.

### Risk stratification

To stratify patients into high- and low-risk groups based on predicted risk scores from the RSF model, Youden’s J statistic was used to identify the optimal cutoff. Youden’s J was calculated as *J = sensitivity + specificity − 1*, using binary event status at a fixed follow-up time.^22^ The cutoff point corresponding to the maximum value of J was selected to define the threshold that best separates patients likely to experience the event from those who are not, this was done on the training set to avoid data leakage. Risk scores were then divided using this threshold, Kaplan-Meier survival curves were used to illustrate the difference in the groups’ survival, and log-rank testing was conducted to assess the statistical difference between them. This step was conducted using the test set to assess the risk stratification on unseen data.

### Clinical application

To assess clinical applicability, Decision Curve Analysis (DCA) was conducted at a horizon of 1 year, using a threshold range from 0 to 0.25 which is generally regarded as a clinically reasonable range for clinical decision-making.^23^ Net-benefit and interventions avoided calculations were conducted and the curves plotted.

An alpha level of 0.05 was selected in this study to indicate statistical significance.

## Results

### Baseline characteristics

Across the full follow-up period, 1235 recipients (18.4%) experienced pancreas graft failure and 5490 (81.6%) did not, a comparison of their baseline characteristics for variables retained in the final dataset after preprocessing and feature selection is presented in Table 1.

**Table 1:** Baseline characteristics of SPK transplant recipients, donors, and surgical factors stratified by graft-failure outcome.

Variable	All (Mean ± SD or %)	No graft failure (Mean ± SD or %)	Graft failure (Mean ± SD or %)	*p*-value
Recipient characteristics				
Age (years)	42.38 (9.18)	42.29 (9.07)	42.79 (9.65)	0.0807
BMI (kg/m^2)	25.82 (3.85)	25.73 (3.82)	26.22 (3.92)	0.0001
Cold Ischemia Time (h)	10.47 (4.62)	10.41 (4.58)	10.70 (4.79)	0.048
Pancreas Preserve Time (h)	10.42 (4.32)	10.36 (4.24)	10.69 (4.63)	0.015
Total Serum Albumin (g/dL)	3.80 (0.64)	3.81 (0.64)	3.75 (0.64)	0.0009
Dialysis: Missing	0.22%	0.16%	0.49%	0.0668
EBV	92.03%	92.35%	90.61%	0.0472
Insulin	4.58%	1.91%	16.44%	0.0000
Insulin: Missing	36.31%	30.86%	60.57%	0.0000
Peripheral vascular disease: missing	1.72%	1.95%	0.73%	0.0043
Type 1 Diabetes	77.68%	77.85%	76.92%	0.5032
Type 2 Diabetes	22.32%	22.15%	23.08%	0.5032
Functional Status: High	15.97%	16.25%	14.74%	0.2053
Functional Status: Medium	80.07%	79.95%	80.65%	0.6038
Functional Status: Low	3.96%	3.81%	4.62%	0.2164
Donor characteristics				
Age (years)	24.33 (8.03)	24.18 (7.93)	25.02 (8.44)	0.0009
BMI (kg/m^2)	23.84 (3.84)	23.79 (3.79)	24.05 (4.05)	0.0345
BUN (mg/dL)	19.38 (13.14)	19.51 (13.03)	18.83 (13.64)	0.1031
CMV	59.39%	58.96%	61.30%	0.1397
ADH	13.17%	13.52%	11.66%	0.09
Donor Sex (male)	70.35%	70.11%	71.42%	0.3817
Ethnic Category: White, Non-Hispanic	59.27%	59.18%	59.68%	0.7731
Ethnic Category: Black, Non-Hispanic	20.15%	19.95%	21.05%	0.4024
Ethnic Category: Hispanic/Latino	17.06%	17.14%	16.68%	0.7289
Ethnic Category: Other	3.52%	3.73%	2.59%	0.0597
Surgical characteristics				
Venous Extension Graft: Missing	0.01%	0.00%	0.08%	0.4139
Graft Placement: Intraperitoneal	86.60%	87.23%	83.81%	0.0016
Graft Placement: Retroperitoneal	7.64%	7.58%	7.94%	0.7126
Graft Placement: Partial Intra/Retroperitoneal	5.75%	5.19%	8.26%	0.0000

BMI, Body Mass Index; EBV, Epstein-Barr virus; BUN, blood urea nitrogen; CMV, cytomegalovirus, ADH, anti-diuretic hormone. ‘Missing’ rows denote binary indicators representing unavailable values in the original registry field.

P values represent univariable comparisons between recipients with and without graft failure during follow-up; continuous variables were compared using Student’s t-test, and categorical variables using chi-square test.

Many characteristics differed significantly between the two groups, namely BMI, kidney cold ischemia time, pancreas preserve time, and EBV status. Insulin use also differed significantly with only 1.91% of the no-graft-failure group and 16.44% of the graft-failure group requiring it (*p* < 0.001). The missingness of data for both Insulin and Peripheral vascular disease was also statistically significantly different between the groups.

For donors, only age and BMI differed significantly between the two groups. Only Graft Placement displayed significant differences among the surgical characteristics. The most common placement being Intraperitoneal at 87.23% in the surviving group and 83.81% in the failed group (*p* = 0.0016).

### Model performance

The final RSF model had a Harrell’s C-index of 0.73 and a Uno’s C-index of 0.58. Time-dependent AUCs for 1 and 2 years were 0.75 and 0.74, respectively, while Brier scores were 0.075 and 0.090, respectively, for the same time periods.

### Risk stratification

**Fig. 1:**
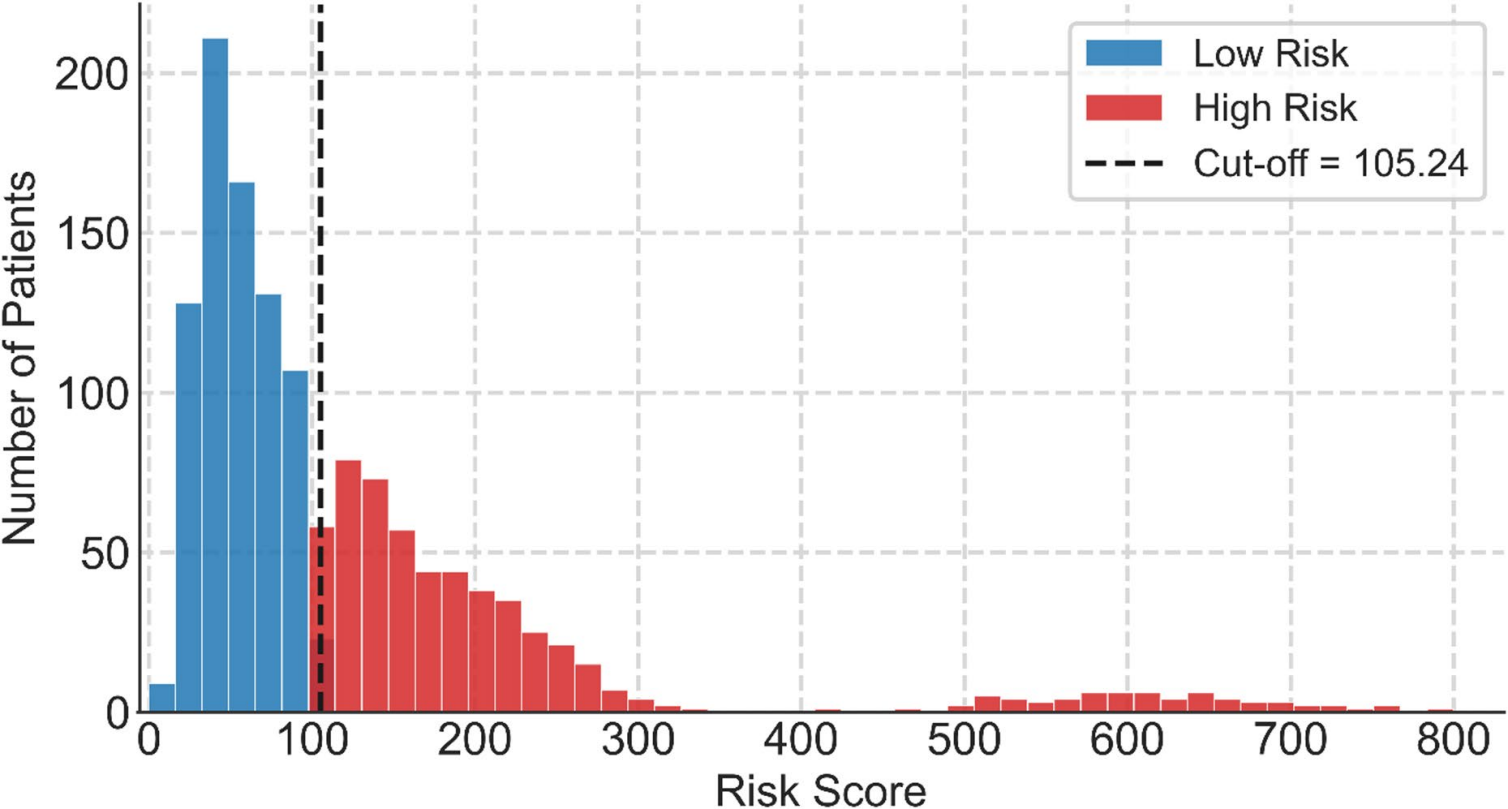
Distribution of individual RSF risk scores with the optimal Youden cut-off (105.24: dashed line).

**Fig. 2:**
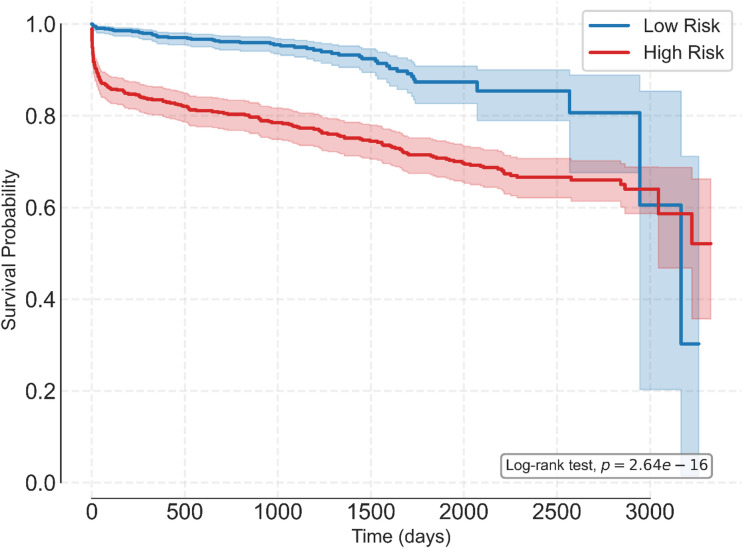
Kaplan–Meier graft-survival curves for low- and high-risk groups.

Risk stratification was conducted by maximizing Youden’s J statistic, which was 0.59, and corresponded to a cut off of 105.24. A histogram of predicted risk scores and the derived threshold is shown in Fig. 1 and a Kaplan-Meier curve showing the different survival curves for each group is shown in Fig. 2.

Risk scores ranged from 9.4 to 814.8 and displayed a right-skewed distribution, with most recipients clustering at lower predicted risk and a smaller subgroup showing substantially higher scores. When the training-derived threshold was applied to the test set, 570 of 1,345 recipients were classified as high-risk and 775 as low-risk.

The low-risk group sustained a higher survival probability throughout most of the period analyzed but experienced a drop in the end due to high censoring. The Log-rank test conducted showed a significant difference (*p* = 2.64e-16) between the two risk groups. This indicates that the RSF-derived threshold meaningfully separated recipients into groups with distinct graft-survival profiles on the independent test set. Estimated survival probabilities at 1 year were 97.2% for the low-risk group and 83.5% for the high-risk group, while 2-year estimated survival probabilities were 96.1% and 80.3% for the low- and high-risk groups respectively.

### Decision curve analysis

**Fig. 3:**
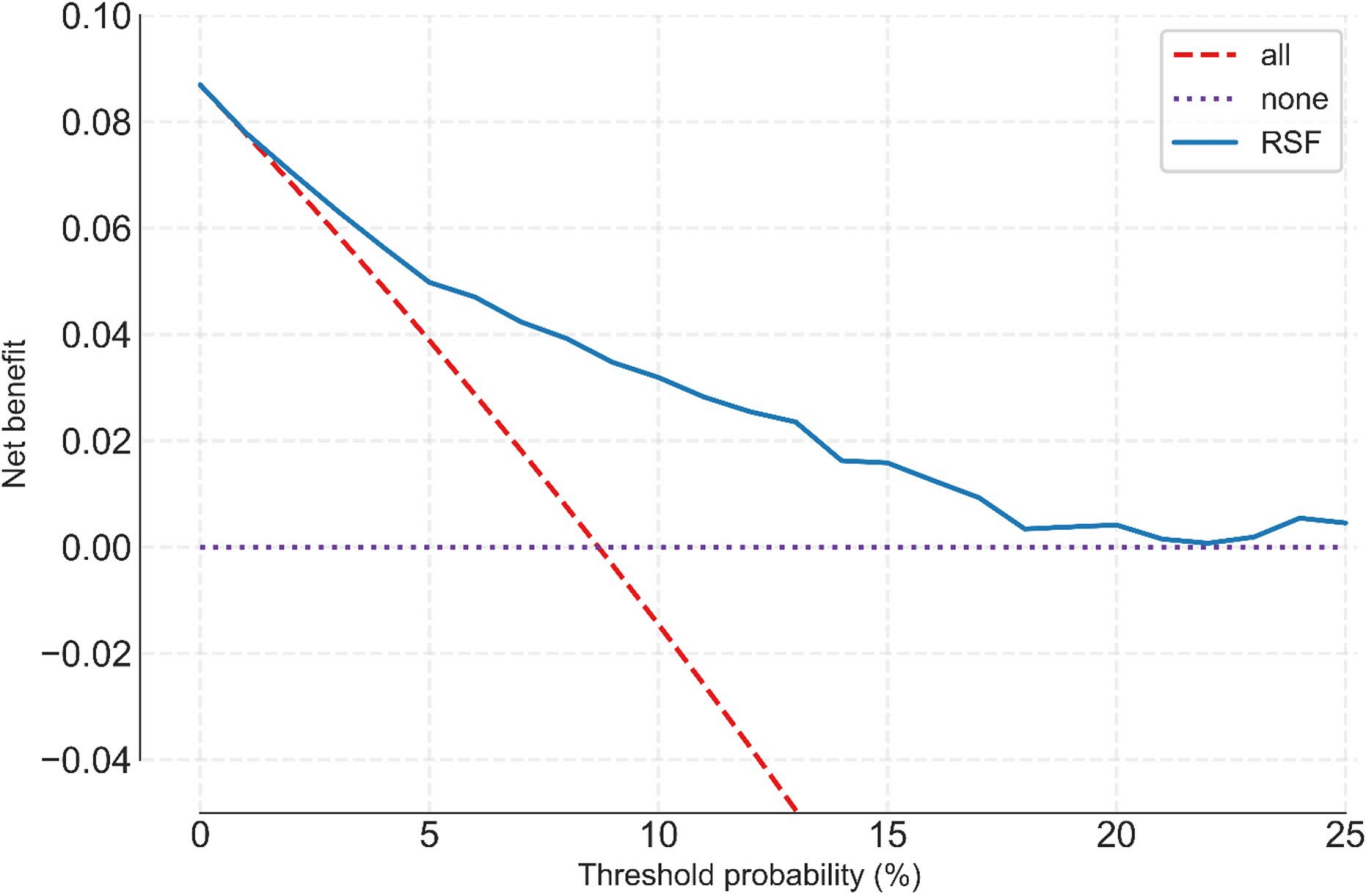
Decision-curve analysis: net benefit of the RSF model versus “treat-all” and “treat-none” strategies across threshold probabilities 0-0.25.

**Fig. 4:**
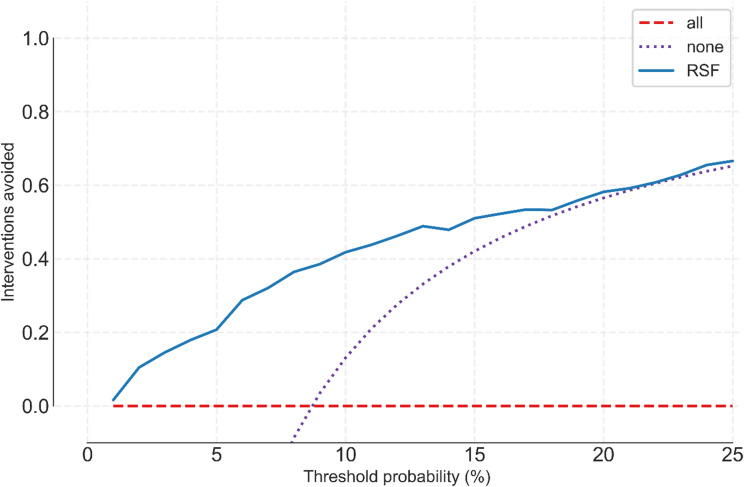
Interventions-avoided curve for the RSF model across threshold probabilities 0-0.25.

To assess clinical applicability and viability DCA was conducted, and the Net-Benefit and Interventions Avoided curves are shown in Figs. 3 and 4 respectively.

At very low thresholds (less than 0.02) the RSF provides a similar benefit to the “treat-all” strategy, but at all other measured thresholds, the RSF provides considerably higher net-benefit compared to the “treat-all” strategy.

The interventions-avoided curve is derived from decision-curve analysis and quantifies how many unnecessary interventions per 100 patients could be avoided by using the model, relative to intervening in all patients, at a given risk threshold. Positive values indicate that the model reduces overtreatment without sacrificing appropriate intervention in patients who truly experience the event.

At every threshold measured, the RSF provides a greater number of interventions avoided compared to the “treat-all” strategy without a drop in the correctly assessed patients in whom intervention is needed. At a threshold of 0.10, for example, the RSF is able to reduce the number of unneeded interventions by 41 without compromising the provision of correct interventions.

**Fig. 5:**
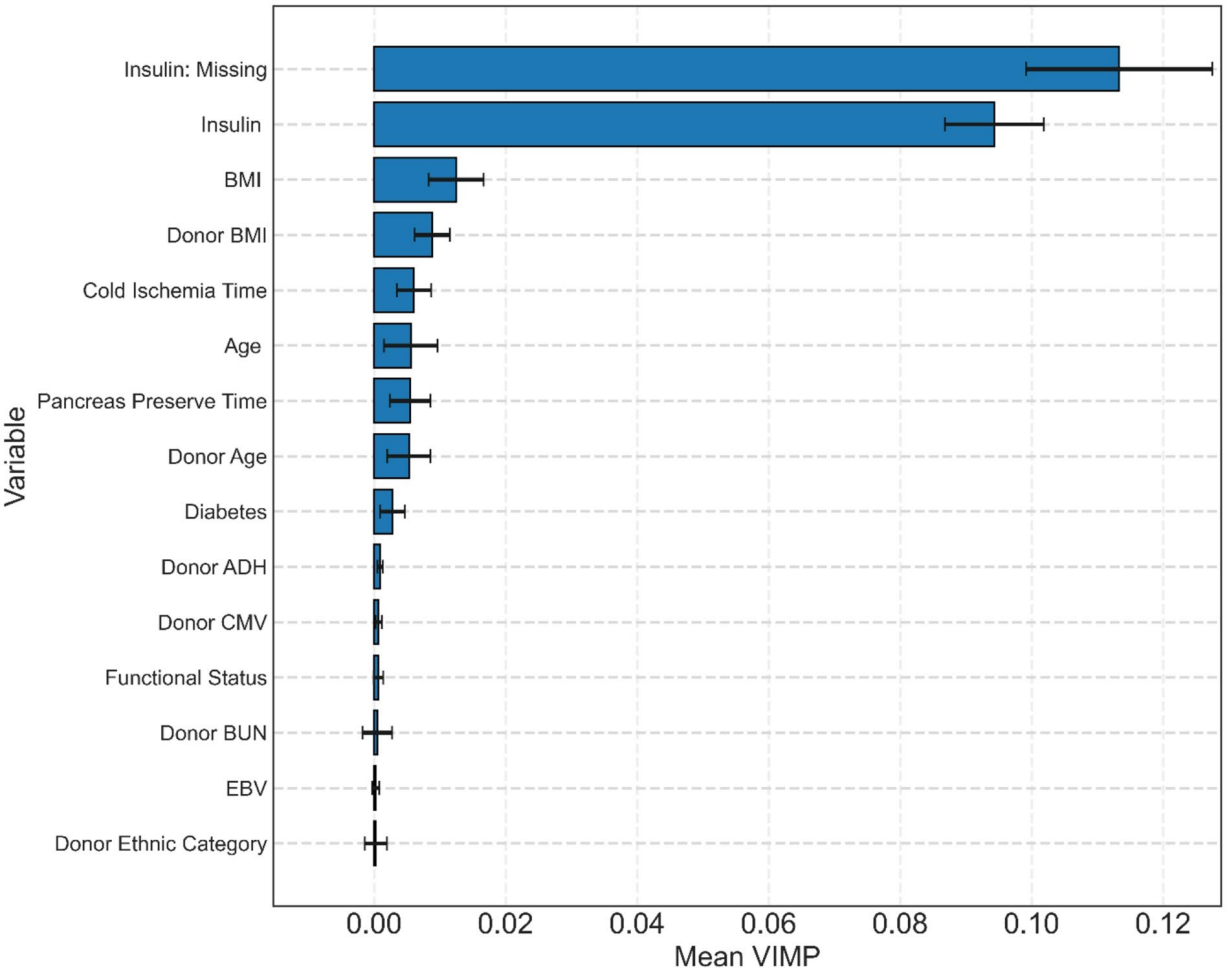
Variable-importance (VIMP) plot for the 15 most influential predictors in the RSF model.

Variable importance analysis is presented in Fig. 5.

Pre-transplant insulin status and its missingness contribute the greatest importance, followed by BMI of the recipient and donor. Cold ischemia time of the kidney, preserving time of the pancreas, as well as both recipient and donor ages were next most important features.

## Discussion

This study focused on developing a machine learning survival prediction model for SPK transplantation. The main predictors utilized by the model were missingness of Insulin usage, Insulin usage itself, cold ischemia time, and recipient age. The patients were classified into low- and high-risk groups whose survival differed in statistically significant manner as shown in Fig. 2.

A majority of the literature concerning machine learning in SPK transplants is focused on binary survival classification of graft failure or rejection at pre-determined time-points, for example, Vigia et al. presented a Naïve Bayes model for graft rejection prediction at 1-year using multiple input features which displayed an AUC of 0.97. Their model ranked long-term insulin usage and the type of dialysis as the most important factors.^10^ Sharma et al. used a Random Forest classifier to predict dnDSA and graft loss using eplet mismatch data, they reported a cross-validated accuracy of 0.639.^24^ Articles using Cox-regression are more common. Rovira et al. presented an immune profiling-based model for predicting acute rejection that showed an AUC of 0.86.^6^ Some articles also focused on measuring the effect of one feature on survival, such as Owen et al., who assessed the effect of the type of diabetes on SPK transplant outcomes and found no significant difference.^8^.

Survival machine learning has yet to be applied to SPK transplantation. It has, however, been utilized in various other solid organ transplants. Kantidakis et al. developed an RSF model to predict survival post liver transplant using UNOS data; they reported a C-index of 0.622 and a Brier score of 0.182.^25^ Liou et al. created a similar model on heart data from the UNOS registry, with their best reported RSF having a C-index of 0.656.^26^ Both of these papers presented reasonable models dealing with similar data to this study. It is worth noting that the C-index reported in both papers is Harrell’s C-index.

In the present study, the RSF achieved a Harrell’s C-index of 0.73 and a Uno’s C-index of 0.58. C-index is measured on a scale of 0 to 1 with 0.5 indicating random chance and 1 indicating a perfect discriminative ability. Harrell’s C-index indicated a good discriminative ability while Uno’s exhibited a better than chance performance. This discrepancy is explained by Harrell’s C-index only comparing pairs in which events occurred, which is greatly impacted by the high censoring rate in the data > 80%, whereas Uno’s implementation uses inverse-probability-of-censoring weighting which was shown to mitigate the over-optimistic values of Harrell’s C-index on heavily right censored data.^21^ A Uno’s C-index of 0.58 is comparable to previous literature conducted by Huang et al., where they analyzed the modeling capabilities of static cox-models and recorded a C-index of 0.56^27^

The C-index, while important for the comparability of models, is not the only metric of interest, as in survival analysis it is imperative that models are also evaluated at clinically relevant time points. Time-dependent AUCs at 1 and 2 years being 0.75 and 0.74, respectively, indicate that the model is reasonably able to classify survival at those points. Brier scores of 0.075 and 0.090 at those respective time points indicate acceptable overall prediction error at those horizons.

Simple evaluation metrics alone are insufficient in measuring the clinical utility of models, therefore both risk stratification analyses and DCA were conducted. Risk stratification is a process which statistically finds the best cutoff to divide the data into high and low risk groups by maximizing Youden’s J, as calculated by Eq. 1. A cutoff of 105.24 (risk score as calculated by the RSF) was determined as the optimal in this study by finding the maximum Youden’s J of 0.59. Estimated survival probabilities were different, with 1-year probabilities of 97.2% and 83.5%, and 2-year probabilities were 96.1% and 80.3%, for the low- and high-risk groups respectively. A Log-rank test (*p* < 0.0001) demonstrated the model’s capability of accurate separation into the groups.

Despite the model’s capability to divide the data in a statistically significant manner into high and low risk groups, clinical utility and significance require further analysis. DCA is a common way to assess whether a test (be that a screening test, a calculated score, or a model risk score) is able to improve patient outcomes by decreasing the number of further interventions required to reach a clinical decision or endpoint, by weighing the cost vs. the benefit when compared to a treat all (high true positives and high false positives) and a treat none (low true positives and low false positives) category.^28^ In this study further treatment or intervention is regarded as increased follow up frequency or increased immune suppressive therapy.

In our analysis a threshold of 0.10 (cost to benefit ratio of 1:9) was chosen.^29^ At that threshold, the use of the risk score output of this model as a screening measure is able to reduce the number of unnecessary interventions by 41 interventions per 100 without a loss of true positive detection compared with a “treat all” approach.

Most pre, post, and intra transplant actions are done to prevent rejection and, by extension, graft failure. Therefore, it is imperative that policies to prevent failure are evidence-based and are reasonably accomplishable by all transplant centers, in spite of their varied resources and capabilities. The current OPTN guidelines for KIDPAN transplants have patients follow-up at 6 months, 1 year, and then annually.^30^.

For example, a recipient classified as high risk based on peri-operative donor, recipient, and preservation variables could undergo closer early surveillance, more frequent biochemical monitoring, or earlier assessment for vascular/technical complications. Conversely, a low-risk patient might remain on standard follow-up pathways. At present, the model should be viewed as an adjunct to peri-operative risk assessment rather than a stand-alone tool for organ acceptance or allocation decisions.

Interpretability in RSF differs from that in conventional regression. Rather than assigning each predictor a single fixed coefficient, the model estimates patient-specific risk from many decision trees and summarizes predictor influence through measures such as variable importance. In practical terms, this means the model can identify which variables most strongly influence predictions while still allowing the estimated effect of a predictor to vary across different clinical contexts.

The most important features as measured by permutation importance were the missingness of data on pre-transplant insulin and transplant insulin itself. This suggests that this variable is of type Missing Not At Random (MNAR), indicating there may be a systematic manner in which this feature goes missing or in its collection, especially since a large percentage of transplant recipients 35.1% are missing this key variable. As this variable (missingness) is not a biologically plausible risk factor in transplants, this draws interest as to whether patient acuity or adherence to the insulin therapy were involved in this process, this is especially interesting since insulin itself, was the second most important predictor. This contrasts published literature by Pham et al. who demonstrated no significant difference in graft survival based on insulin usage in SPK transplants. Vigia et al., on the other hand, reported insulin use to be one of the most predictive features.^10^ Our analysis showed a significant difference between the two groups, with 1.91% of non-event patients on insulin and 16.44% of those undergoing insulin therapy pre-transplant. This difference may be caused by the different methods of classifying insulin usage. Pham et al. utilized units per day at a binary cut-off of 75 as well as a units per kg per day calculation at a cut-off of 0.5. OPTN data, on the other hand, uses a binary YES/NO insulin used. This is more biologically plausible as patients requiring insulin tend to have more complicated/difficult conditions as opposed to those who do not require insulin.^2^.

Other key features were cold ischemia time for the kidney and the age of the recipient, which are generally similar to many other transplant types. As demonstrated by Lum et al. is the cases of cold ischemia time and by Valdiva et al. for recipient age. These factors are also quite biologically plausible as organs that spend more time in an ischemic state are expected to provide worse outcomes. A recipient’s age is associated with many risk factors, the main one being the many comorbidities they often exhibit. ^31,32^ Both recipient and donor BMI were also among the top features. This correlates with the findings of Jarrar et al., who demonstrated the effect of donor-recipient obesity on graft loss in kidney transplantation and found significantly higher risk for loss outcomes when compared to non-obese donor and recipient pairs.^33^ Bellini et al. focused on recipient obesity and found an impact on graft function and graft survival in kidney transplant recipients.^34^.

### Limitations

This study dealt with a few limitations which must be considered when applying its findings to diverse contexts. Namely, this study focused solely on SPK transplants, which, although they represent the majority of pancreatic transplants currently, do not represent them all. Furthermore, the limiting of the study’s time frame to the 2014–2024, reduced the maximum follow-up time and introduced many recent transplants. Also, although geographically diverse, the study focuses entirely on transplants done in the USA under OPTN guidelines for matching and follow-up, which do differ across the globe and may impact the generalizability of the model. Additionally, missing data represented quite a large issue both in terms of imputation as well as the exclusion of potentially useful features due to their recent introduction in the database and lack of data on older transplants. Moreover, our model required the use of donor and surgical characteristics, which means that it may best function as a peri-transplant model, as factors such as duct management or cold ischemia time may not be available pre-transplant.

Furthermore, the model was evaluated using a single random train-test split. Although this preserved an untouched holdout set for internal validation, it does not capture the variability in model performance that may arise across different random partitions of the data. Accordingly, the reported results should be interpreted as internal holdout validation rather than a definitive estimate of generalizable performance. Repeated resampling and external validation are required before clinical implementation.

## Conclusions

Machine learning tools should aid and support and not replace clinical decision making by physicians. This study presents a promising internally validated model for risk stratification after SPK transplantation that may support peri-operative risk assessment and follow-up planning. Further research must focus on determining the optimal “intervention” or additional measures taken in the care of patients classified into the high-risk group, necessarily by determining the causes of graft failure in those patients and assessing different follow-up schedules and therapeutic policies. Prospective and external validation are required to establish the model’s clinical utility and generalizability.

## Data Availability

The data used in this study were obtained from the Organ Procurement and Transplantation Network (OPTN) / United Network for Organ Sharing (UNOS) through the OPTN data request process under a Data Use Agreement and are not directly publicly downloadable. Researchers may request access to the same dataset through the OPTN Data Request process at: [https://optn.transplant.hrsa.gov/data/request-data/](https:/optn.transplant.hrsa.gov/data/request-data) . Access is subject to application review, approval, and applicable data-use conditions for secondary analysis.
